# Poster 2010: A study assessing the safety and efficacy of subcutaneous immunotherapy with a dust mite vaccine with the adjuvant l-tyrosine in a highly sensitized population in a Latin-American city

**DOI:** 10.1186/1939-4551-7-S1-P22

**Published:** 2014-02-03

**Authors:** Ricardo Cardona Villa, Carlos Fernando Chinchilla Mejia, Ruth Helena Ramirez Giraldo, Maria Margarita Olivares Gomez, Victor Calvo, Jorge F Maspero

**Affiliations:** 1 Universidad de Antioquia, I.P.S Universitaria-Clinica León XIII, Medellin, Colombia; 2 IATM, Medellin, Colombia; 3 Universidad de Buenos Aires, Fundacion Cidea, Buenos Aires, Argentina

## Background

In Medellín-Colombia dust mite allergy is prevalent with high intensity of sensitization. We intended to describe the tolerability and efficacy of a new vaccine with a new adjuvant L-tyrosine in this population.

## Methods

We realized a 2 years ambispective study for assessment safety and efficacy **of subcutaneous immunotherapy with a dust mite vaccine with the adjuvant L-tyrosine**. We reviewed the data of 292 patients with allergic rhinitis and/or asthma. They are sensitized to *Dermatophagoides pteronyssinus or Dermatophagoides farinae and or Blomia tropicallis*. A data base was constructed to allow complete assessment of the characteristics of the patients. We used the grading system proposed by the World Allergy Organization for the classification of the systemic reaction.

## Results

### Safety

The subjects received a total of 3760 doses and 40 systemic adverse reactions were registered (reaction rate 1.06%). Of this 40 systemic reactions, 34 were in different patients and 6 patients had 2 reactions each. 28 reactions were grade 1, 2 and only one reaction was graded 3 and all were kept under monitoring until complete recovery from the event. 11 reactions were grade 1 and resolved with antihistamines alone (1z). No patients died and none patient was admitted or required ICU or intubation. Reactions were more common in patients with allergic rhinitis (57%) than in asthmatics (30.8%). 90 % of the reactions occurred with the allergen vaccine with mixture of *D. pteronyssinus plus D. farinae 50%*+*50 %* (16.666 TU/0.5 ml).

### Efficacy

86.3 % of the patients improved markedly their clinical scores and or medication requirements, the response were partial for 7.1 % and absent for 6.7 %. **The efficacy was assessment by medical criteria and was grater in those patients that don't had systemic adverse reactions in contrast to whom had systemic reactions (88.9 vs. 66.7%)**.

## Conclusions

Subcutaneous Immunotherapy with L-tyrosine adjuvant for dust mite allergens is safe and effective in a highly sensitized population in a Latino-American Tropical City.

